# Kaempferol is a novel antiviral agent against channel catfish virus infection through blocking viral attachment and penetration *in vitro*

**DOI:** 10.3389/fvets.2023.1323646

**Published:** 2023-12-04

**Authors:** Kai Hao, Yu Wang, Jie Hua Xu, ChunLan Nie, SiYang Song, Fei Yu, Zhe Zhao

**Affiliations:** Jiangsu Province Engineering Research Center for Marine Bio-resources Sustainable Utilization, College of Oceanography, Hohai University, Nanjing, China

**Keywords:** channel catfish virus, kaempferol, traditional Chinese medicine, antiviral activity, antiviral mechanism

## Abstract

Channel catfish virus (CCV, *Ictalurid herpesvirus* 1) is the causative pathogen of channel catfish virus disease, which has caused high mortality and substantial economic losses in the catfish aquaculture industry. Due to the lack of licensed prophylactic vaccines and therapeutic drugs, the prevention and control of CCV infection seem to remain stagnant. Active compounds from medicinal plants offer eligible sources of pharmaceuticals and lead drugs to fight against endemic and pandemic diseases and exhibit excellent effect against viral infection. In this study, we evaluated the antiviral ability of 12 natural compounds against CCV with cell models *in vitro* and found kaempferol exhibited the strongest inhibitory compound against CCV infection among all the tested compounds. Correspondingly, kaempferol decreased transcription levels of viral genes and the synthesis of viral proteins, as well as reduced proliferation and release of viral progeny, the severity of the CPE induced by CCV in a dose-dependent manner, based on quantitative real-time PCR (RT-qPCR), western blotting, viral cytopathic effects (CPE) and viral titer assessment. Moreover, time-of-drug-addition assays, virus attachment, and penetration assays revealed that kaempferol exerted anti-CCV activity probably by blocking attachment and internalization of the viral entry process. Altogether, the present results indicated that kaempferol may be a promising candidate antiviral agent against CCV infection, which shed light on the development of a novel and potent treatment for fish herpesvirus infection.

## Introduction

Aquatic products are a vital part of the global food supply and occupy an important position in economic industry worldwide (1). However, frequent outbreaks of infectious diseases (bacteria, fungi, viruses, or parasites) have now become a key issue affecting the aquaculture industry (2–4). Among the pathogens, viruses are probably the most devastating in the aquaculture industry. Channel catfish virus (CCV) is an enveloped, double-stranded DNA virus of the family *Alloherpesviridae*, and can provoke fatal infections in fingerling channel catfish (5). Due to the high-density fish culture, the deterioration of the farming environment, and the degradation of germplasm, outbreaks of CCV infection are progressively increasing, which seriously hamper the development of channel catfish culture (6, 7). To date, no commercially approved vaccine against CCV is available around the world. To make matters worse, there are still no available and effective drugs to treat CCV-infected fish. It is therefore necessary and urgent to develop novel antiviral drugs against CCV disease.

Traditional Chinese medicine, including crude plant extracts, natural compounds, and their analogs (8), act as important sources of therapeutic agents and have been frequently applied in the diseases treatment of humans, poultry, and livestock with high bioactivity and bioavailability as well as minimal side effects (9–11). For example, it was found that the natural compounds resveratrol, epigallocatechin gallate, and quercetin, which belonged to the polyphenols, exhibited significant antiviral activity against severe acute respiratory syndrome coronavirus, Zika virus, and dengue virus (12–15). In recent decades, phytomedicines have also been widely used in aquaculture and some natural compounds have been reported against aquatic animal viral diseases (8). For instance, for grass carp hemorrhagic disease, magnolol and honokiol extracted from *Magnolia* had a significant inhibitory effect on the replication of grass carp reovirus *in vitro* and *in vivo* via activating the host antiviral immune response (16, 17). Meanwhile, the natural compound coumarin and its analogs play a critical role against aquatic rhabdovirus infection (18–20). It is worth mentioning that current research on anti-herpesvirus natural drugs is relatively lacking in aquaculture. Crude plant extracts from *Arthrospira platensis* and *Clinacanthus nutans* had antiviral effects against koi herpesvirus (21). The anisodamine extracted from the solanaceae plant and berberine extracted from *Berberis* genus have been reported to have anti-cyprinid herpesvirus 2 activity (22). The above research results have attracted attention to the screening and discovery of novel anti-CCV candidates from natural substances. In addition to effective antiviral activity, the use of phytomedicines can reduce large-scale abuse of chemicals and antibiotics in fish farming and avoid the occurrence of a variety of potential risks, including antibiotic residues and resistances (23), which provide a promising alternative approach for controlling fish viral diseases.

Kaempferol is a kind of flavonoid polyphenol compound and has various biological and pharmacological properties, including antimicrobial, anticancer, and antioxidant (24, 25). In the past decade, the anti-viral ability of kaempferol has been well investigated in human diseases. Several studies indicate that kaempferol displays potent as well as broad antiviral activity against herpesvirus (26), hepatitis virus (27), influenza virus (28), and coronavirus (29, 30). However, there has been little research on the anti-viral effects of kaempferol in aquaculture. Interestingly, some other flavonoids have been studied widely and exhibit antiviral effects on a variety of aquatic viruses. For instance, curcumin inhibits Singapore grouper iridovirus (SGIV) infection through multiple antiviral mechanisms (31). The fisetin and fustin extracted from *Rhus verniciflua* bark exert high antiviral activity against infectious hematopoietic necrosis virus and viral hemorrhagic septicemia virus (32). Therefore, these studies offer a range of options for developing antiviral herbal medicine for treating and preventing CCV infection in channel catfish farming.

In the present study, we evaluated the anti-CCV potentials of 12 natural compounds and identified that kaempferol exhibited prominent antiviral ability compared with other tested compounds using the CCO cells infection model. We further confirmed the inhibitory effects of kaempferol for CCV infection *in vivo*. Mechanistically, we showed that kaempferol blocked CCV entry, possibly through interacting with the adsorption and internalization of CCV. Our results highlight the indispensable anti-CCV activity of kaempferol and provide a potential antivirus agent for preventing CCV infection.

## Materials and methods

### Cell, virus, and compounds

The channel catfish ovary (CCO) cells were cultured in Dulbecco’s modified Eagle’s medium (DMEM Gibco, USA) containing 10% fetal bovine serum (FBS, Gibco, USA), hepes buffer, penicillin (100 IU/mL) and streptomycin (100 μg/mL) (Gibco, USA) at 28°C. The CCV (strain VR-665) was propagated in CCO cells and the viral titers were performed with 50% tissue culture infective dose (TCID_50_) assays (33). The tested compounds were purchased directly from commercial companies. The CAS number and purities of all compounds are listed in Supplementary Table S1. All compounds were dissolved in DMSO (Sigma) and stored at −20°C for use within 6 months.

### Compounds cytotoxicity assay

The cytotoxicity effects of the selected compounds were detected in CCO cells by the cell viability assay. In brief, the CCO cells were seeded in 96-well plates and cultured to reach approximately 90% confluence in each well at 28°C. Subsequently, the monolayer cells were exposed to a maintenance medium with the tested compounds at different concentrations or DMSO for 72 h. Cell Counting Kit-8 (Beyotime, China) was used to measure the cell viability according to the manufacturer’s protocol after 72 h incubation. The optical density at 450 nm was measured using a Spark microplate reader (TECAN, Switzerland). Then, the maximum safe concentration (≥ 90% cell survived, SC_90_) of compounds to CCO cells was calculated according to the cell viability.

### Antiviral activity screening assay of compounds

To test the anti-CCV effect of tested compounds in the preliminary screening, CCO cells were seeded in 24-well plates and cultured to reach approximately 90% confluence in each well at 28°C. Subsequently, the cells were infected with the CCV at 10^3^ TCID_50_/ml at 28°C for 1 h. After that, the cell culture medium was replaced using an isopycnic DMEM medium containing the maximum safe concentration of indicated compounds. CCV-infected cells treated with DMSO served as a negative control, whereas CCV-infected cells treated with acyclovir (2.5 μg/mL) served as a positive control (33). After 36 h treatment, cell lysates were collected to test the CCV gene expression by the RT-qPCR, and the percentage inhibition of CCV infection by individual compound was calculated. According to the percentage inhibition results, we further detected the antiviral activity of kaempferol at maximum safe concentration by viral cytopathic effects (CPE) and western blot analysis.

### Anti-CCV replication assay of kaempferol

The CCO cells were infected with the CCV at 10^3^ TCID_50_/ml at 28°C for 1 h. Then, the cell culture medium was replaced using isopycnic DMEM medium containing various concentrations kaempferol (2.5, 5, 7.5, 10, 12.5, and 15 μg/mL). The cells without exposure to kaempferol and CCV were set as the mock control. CCV-infected cells served as a negative control. 24 and 36 h post-infection, the cells were collected and subjected to the RT-qPCR and western blot analysis for the viral gene and protein expression. Meanwhile, the cells were treated as described above for the determination of virus titer. The supernatant and cell pellets were collected at 24 and 36 h post-infection and the TCID_50_ was used to evaluate the viral loads of intracellular and extracellular.

### CPE reduction and liquid plaque assay

For analyzing the CPE reduction of viral infected CCO cells by kaempferol, the CCO cells were seeded in 24-well plates and cultured overnight at 28°C before infection. Then, the monolayer cells were infected with CCV at 10^3^ TCID_50_/ml for 1 h. Subsequently, the infected cells were treated with kaempferol at different concentrations (2.5, 5, 7.5, 10, 12.5, and 15 μg/mL), respectively. The CCO cells without CCV and drug treatment were set as mock control whereas the virus-infected cells without compound treatment were set as CCV control. Each sample was directly observed and photographed under an inverted microscope at 24 and 36 h post-infection. Meanwhile, the plaque assay was performed as described in previous studies with slight modifications (34). Briefly, confluent cells in 24-well plates were infected with CCV (10^3^ TCID_50_/ml) for 1 h. Subsequently, the inoculums were removed and the kaempferol with different concentrations (2.5, 5, 7.5, 10, 12.5, and 15 μg/mL) was added and remained throughout the experiment. The cells were fixed and stained with 1% crystal violet and then photographed at 36 h post-infection.

### Time-of-drug-addition assay

The CCO cells seeded in 24-well plates were infected with CCV under five different treatment conditions: (i) Control: CCO cells cultured in the maintenance DMEM media were infected by CCV for 1 h. (ii) Pre-treatment: CCO cells were pre-treated with 15 μg/mL of kaempferol for 2 h before CCV infection. (iii) Virus treatment: CCV was pre-incubated with kaempferol at 37°C for 2 h before being added to CCO cells. (iv) Virus and kaempferol co-treatment: CCO cells were treated in media containing both kaempferol and CCV at 28°C for 1 h. (v) Post-treatment: CCO cells were incubated with CCV at 28°C for 1 h. After that, kaempferol (15 μg/mL) was supplemented at 1, 2, 4, 8, 12, and 24 h post-infection. In all treatment conditions, the unabsorbed CCV was washed off twice by PBS after 1 h of adsortion. The samples were collected to test the CCV gene expression by the RT-qPCR at 24 h and 36 h after the corresponding treatments.

### Virus attachment assay

The attachment assay was performed as previously described, with slight modifications (35). Briefly, monolayer CCO cells seeded in 24-well plates were pre-cooled at 4°C for 30 min, and then the pre-cooled cells were treated with kaempferol (15 μg/mL) or left untreated for 2 h at 4°C. Cells were then incubated with CCV at 4°C for 1 h to allow virus attachment but prevent viral internalization. Thereafter, the compound and unattached virus were washed with ice-cold PBS and a fresh DMEM medium was added. After incubated at 28°C for 24 h, the cells were collected for subsequent experiments, including RT-qPCR and viral titer.

### Virus penetration assay

Confluent CCO cells in 24-well plates were prechilled at 4°C, and then infected with CCV at 4°C for 1 h. After triple washing with pre-cooled PBS, the cells were treated with kaempferol (15 μg/mL) or medium at 28°C for 4 h. The compound dilutions were aspirated and the cells were rinsed with citrate buffer (pH 3.0) in order to inactivate and remove not penetrated virus. After incubated at 28°C for 24 h, the cells were collected for subsequent experiments, including RT-qPCR and viral titer.

### Virus titration assay

Measurement of viral titer was described previously (5). In brief, CCO cells were cultured in 96-well plates, and they were infected with 10-fold serially diluted samples that were collected previously. At 72 h post-infection, the cells were directly observed under an inverted microscope. The TCID_50_ was used to evaluate the viral titers at the indicated times.

### RNA isolation and RT-qPCR analysis

For RT-qPCR, the total RNA was isolated from CCV-infected cells using RnaExTM (Trizol)^a^ reagent (GENERAY, China), and reversed transcribed applying a HiScript III RT SuperMix with gDNA wiper (Vazyme, China) following the manufacturer’s instructions. Quantitative PCR was performed with a LightCycler 96 Real-Time PCR Detection System (Roche, USA) using ChamQ Universal SYBR qPCR Master Mix (Vazyme, China) with the following cycle conditions: 95°C for 30 s, followed by 40 cycles of 95°C for 10 s and 60°C for 30 s. To assess the specificity of each amplicon, melt curve analysis was also performed at the end of each thermal profile. The primers used for PCR are listed in Supplementary Table S2. Each individual sample was run in triplicate wells. Fold changes of the selected gene vs 18 s (internal reference) were calculated by the 2^−△△^Ct method.

### Western blotting analysis

For the western blot assay, firstly, cells were lysed with cell lysis buffer (Beyotime, China), and then the concentrations of protein samples collected from cell lysates protein were determined using the Enhanced BCA Protein Assay Kit (Beyotime, China). Equal amounts of proteins were separated by 12% SDS-PAGE gels and transferred onto the PVDF membrane (Millipore, USA). The PVDF membranes were then blocked for 2 h at room temperature with 5% skim milk and washed three times with PBST (0.05% Tween in 0.01 M PBS). The PVDF membranes were probed with primary antibody comprising rabbit anti-CCV ORF 39 and ORF 59 antibody (prepared by our laboratory) (7) and mouse monoclonal anti-GADPH (Sigma) was used as a control. Then the membranes were washed with PBST buffer and incubated with secondary antibody (HRP goat anti-rabbit or goat anti-mouse IgG, Sigma) for 2 h. Finally, the signals were detected using an ECL kit (Beyotime, China) according to the manufacturer’s instructions.

### Statistical analysis

All data were analyzed with GraphPad Prism 8.0. Viral titers were log_10_ transformed prior to statistical analyses. The SC_90_ values were calculated using probit analysis. The data are expressed as the arithmetic mean ± standard deviation (SD) and were analyzed by one-way ANOVA with multiple comparisons test. Statistical significance was defined as *p < 0.05, **p < 0.01.

## Results

### Screening for the anti-CCV natural compounds in CCO cells

**Figure 1:**
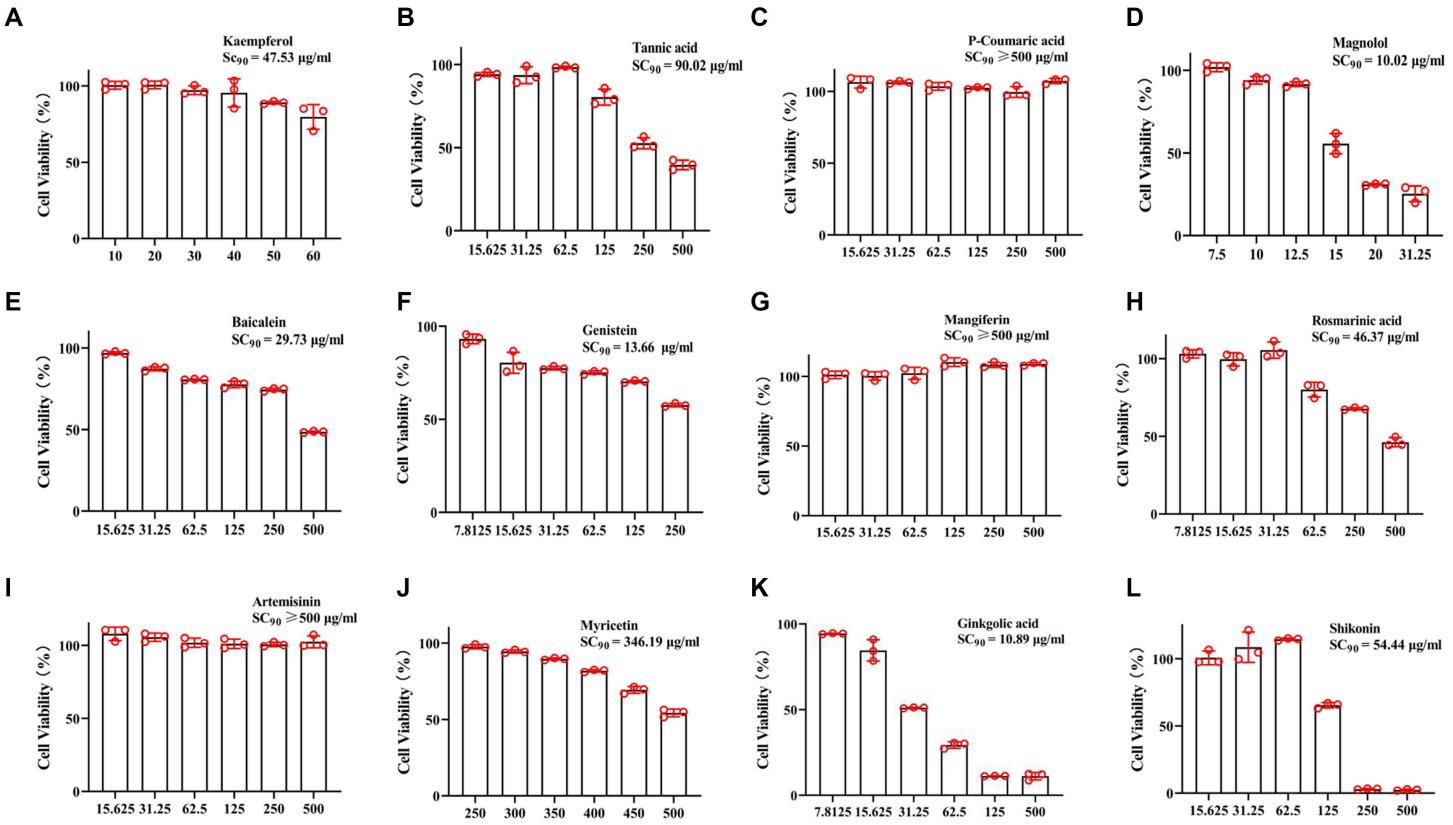
Cytotoxicity assay. The CCO cells were treated with 12 tested compounds with different concentrations in a 96-well plate for 72 h, and then the cell viability was measured by CCK-8. **(A)** Kaempferol, **(B)** Tannic acid, **(C)** P-Coumaric acid, **(D)** Magnolol, **(E)** Baicalein, **(F)** Genistein, **(G)** Mangiferin, **(H)** Rosmarinic acid, **(I)** Artemisinin, **(J)** Myricetin, **(K)** Ginkgolic acid, **(L)** Shikonin. Data represent mean ± SD normalized to values for DMSO treated group. The SC_90_ was analyzed and calculated using probit analysis.

**Figure 2:**
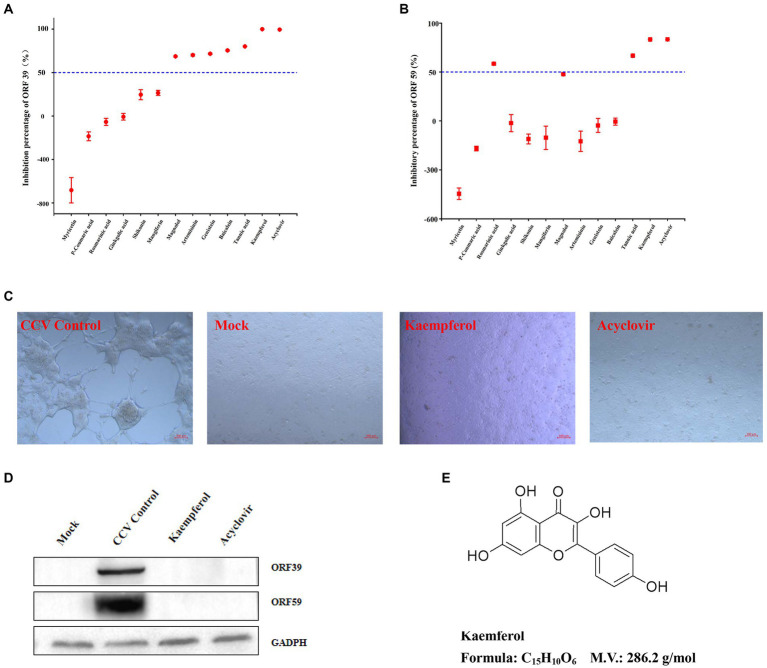
The anti-CCV activity preliminary screening assay of the compounds. **(A,B)** The CCO cells infected by CCV were treated with 12 tested compounds or acyclovir (positive control), and samples were collected at 36 h post-infection for the detection of CCV ORF 39 and ORF 59 genes expression levels by RT-qPCR. The percent inhibition of CCV ORF 39 and ORF 59 by the tested compounds were calculated and expressed as the mean ± SD. **(C)** The CCO cells were treated with kaempferol at the maximum safe concentration or acyclovir after CCV infection 1 h, then, the morphology of CCO cells was observed by light microscopy at 36 h post-infection. **(D)** The CCO cells were treated with kaempferol at the maximum safe concentration or acyclovir after CCV infection 1 h, then, cells were collected 36 h after infection and CCV ORF 39 and ORF 59 proteins and cellular GADPH were analyzed by western blot. **(E)** Chemical molecular structure of kaempferol.

Natural compounds from traditional Chinese medicine have been considered valuable sources for antiviral treatments. To this end, the anti-CCV effects of 12 compounds (listed in Supplementary Table S1) were tested. First, the safe concentration (≥ 90% cell survived, SC_90_) of compounds was estimated in CCO cells by the cell viability test (Figure 1). The inhibition rate of the CCV ORF 39 and 59 genes was calculated for screening the compounds with strong antivirus activity. Acyclovir treatment was used as a positive control as previously reported (33). Notably, kaempferol exhibited excellent inhibition on virus reproduction with an inhibitory rate of 99.9%, which showed an equivalent anti-CCV activity to acyclovir (Figures 2A,B). Therefore, we focused on kaempferol for further study. CPE observation and western blot analysis further showed that kaempferol significantly reduced CPE and suppressed the expression of viral proteins induced by CCV infection at the maximum safe concentration of the compound (47.53 μg/mL) (Figures 2C,D). Present results suggested the potential use of kaempferol (Figure 2E) as a novel anti-CCV infection compound.

### Kaempferol exhibited potently antiviral activity with dose-dependent *in vitro*

**Figure 3:**
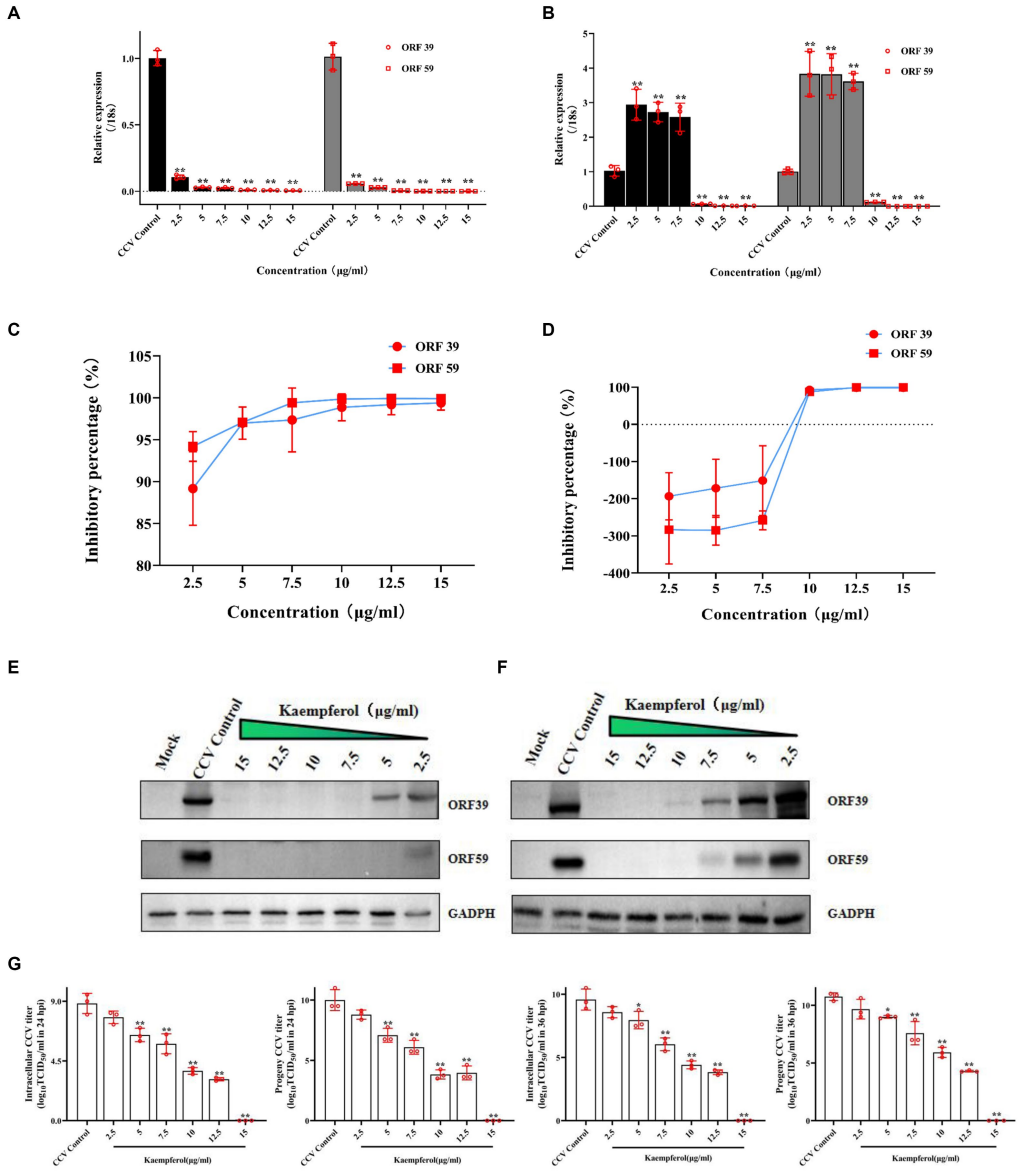
The anti-CCV activity by different concentration of kaempferol *in vitro*. The CCO cells were infected with the CCV for 1 h. Then, the cells were incubated with different concentrations of kaempferol (2.5, 5.0, 7.5, 10, 12.5 and 15.0 μg/mL).The cells without additional treatments were set as the mock control. Only CCV-infected cells served as the CCV Control. The relative expression levels of the CCV ORF 39 and ORF 59 genes were evaluated by RT-qPCR at 24 h **(A)** and 36 h **(B)** post-infection. The selected gene level was normalized to 18 s (internal reference) using the 2^− ΔΔCT^ method. The percent inhibition of CCV ORF 39 and ORF 59 by the kaempferol was calculated at 24 h **(C)** and 36 h **(D)** post-infection, respectively. The CCO cells were infected with the CCV for 1 h. Then, the cells were incubated with different concentrations of kaempferol (2.5, 5.0, 7.5, 10, 12.5 and 15.0 μg/mL). The cells without additional treatments were set as the mock control. Only CCV-infected cells served as the CCV control. The cells were collected at 24 h **(E)** and 36 h **(F)** after infection and CCV ORF 39 and ORF59 proteins and cellular GADPH were analyzed by western blot. **(G)** The intracellular and released CCV virions separated from cell lysates and supernatants at 24 h and 36 h post infection were analyzed by viral titer. Data represent mean ± SD. The *p* value was determined by *p < 0.05, **p < 0.01.

Kaempferol exhibited the most potent activity against CCV infection among all tested compounds *in vitro* as described above, resulting in its selection for further study on its anti-CCV activity. First, RT-qPCR data revealed that kaempferol treatment markedly decreased the expression of CCV ORF 39 and CCV ORF 59 with the gradient increase in kaempferol concentration (Figures 3A,B). At 24 h post viral infection, The inhibitory effect of CCV-ORF 39 and CCV-ORF 59 by kaempferol over 85% (Figure 3C). At 36 h post viral infection, viral mRNA levels still were significantly lower compared with the control group when the kaempferol concentration ranged from 10 μg/mL to 15 μg/mL (Figures 3B,D). Consistent with the results of RT-qPCR, the protein level of CCV ORF 39 and ORF 59 decreased as the concentration of kaempferol increased. Notably, the viral protein in kaempferol treatment remained at a nearly undetectable viral loading when kaempferol concentration was from 10 μg/mL to 15 μg/mL at both 24 and 36 h (Figures 3E,F). The anti-CCV activity of kaempferol was further verified with a viral titer assay. The results showed that kaempferol dramatically reduced intracellular and progeny viral loads in response to an increase of compound concentration at both 24 and 36 h post CCV infection (Figure 3G). Collectively, these results further confirmed that kaempferol has excellent antiviral activity against CCV in a dose-dependent manner.

### Kaempferol improved morphological protective effect on CCV infected CCO cells

Virus-induced CPE was observed under treatment with different kaempferol concentrations.

**Figure 4:**
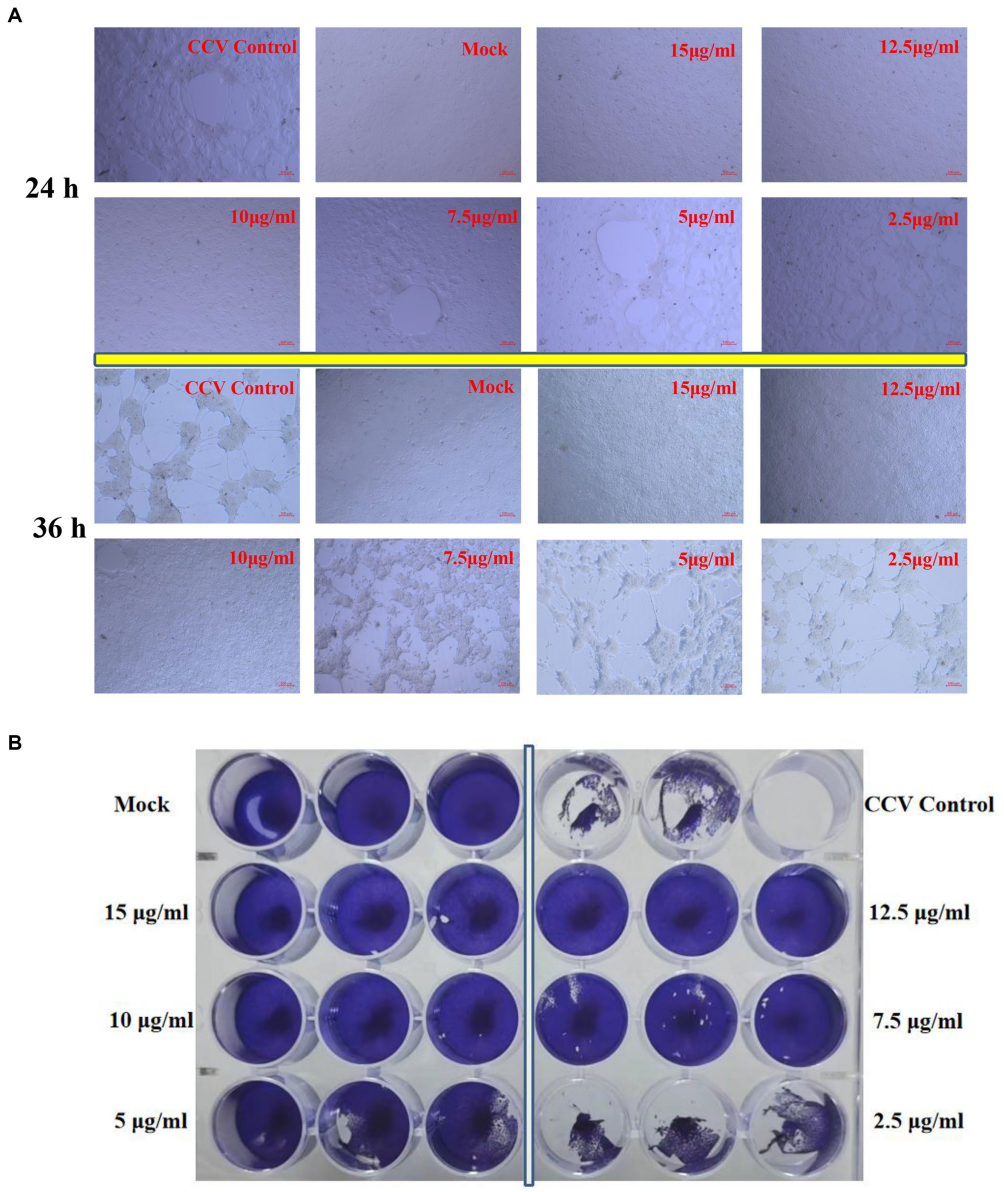
CPE and plaque reduction in CCO cells treated with kaempferol. **(A)** The cells were incubated with different concentrations of kaempferol (2.5, 5.0, 7.5, 10, 12.5 and 15.0 μg/mL) after CCV infection 1 h. Afterwards, the severity of the CPE induced by CCV was directly observed and photographed under an inverted microscope at 24 and 36 h post-infection. **(B)** The cells were treated with CCV for 1 h. Subsequently, the kaempferol with different concentrations (2.5, 5, 7.5, 10, 12.5 and 15 μg/mL) was added for 36 h. The cells were fixed and stained with 1% crystal violet and then photographed.

The untreated cells infected with CCV cells acquired an obviously cytopathic effect at 24 h, and large amounts of cell death occurred sharply within 36 h (Figure 4A). In contrast, Exposure to kaempferol protected CCO cells against CCV-induced CPE as the concentration of kaempferol increased. Exhilaratingly, no obvious CPE was observed until 36 h in kaempferol treatment with 10–15 μg/mL concentration (Figure 4A). Accordingly, the inhibition of viral plaque formation was substantially reduced from the infected CCO cells upon kaempferol treatment by liquid plaque assay, while kaempferol at 10 μg/mL still showed close to 100% inhibition of viral plaque formation (Figure 4B).

### Kaempferol exhibited an effect on the CCV entry process

**Figure 5:**
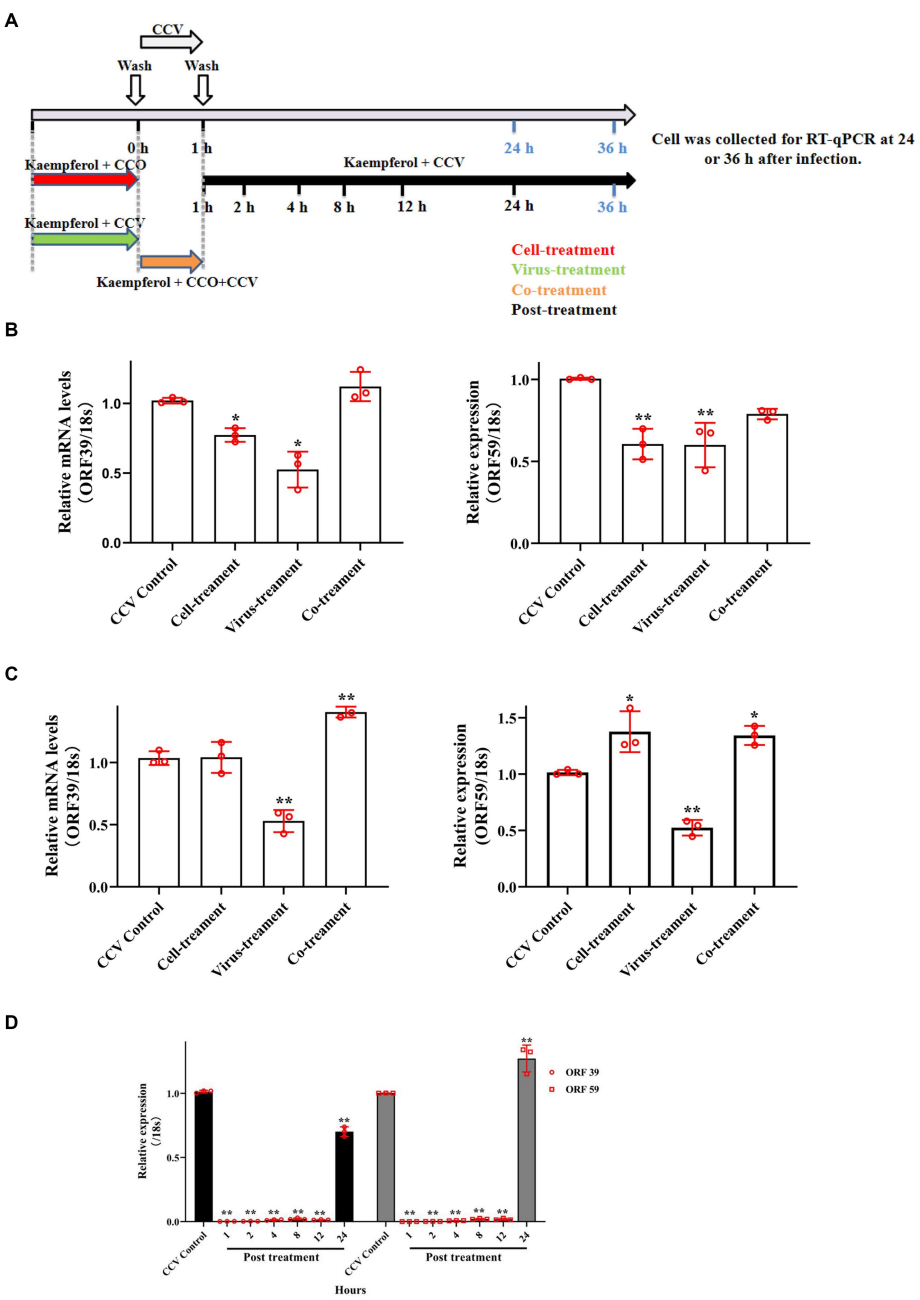
Time of drug-addition assay of kaempferol in CCV inhibition. **(A)** The schematic diagram of the experimental settings. The CCO cells were infected with CCV for 1 h followed by the indicated kaempferol treatments. Cell lysates at indicated time post-infection were subjected to RT-qPCR. **(B,C)** The relative expression levels of the CCV ORF 39 and ORF 59 genes with cell-treatment, virus-treatment and co-treatment at 24 h and 36 h post-infection. **(D)** The relative expression levels of the CCV ORF 39 and ORF 59 genes with post-treatment at 36 h post-infection. The selected gene level was normalized to 18 s (internal reference) using the 2^− ΔΔCT^ method. Data represent mean ± SD. The *p* value was determined by *p < 0.05, **p < 0.01.

To further explore the potential antivirus mechanisms of kaempferol against CCV, time-of-drug-addition assay was performed to investigate which stage of the viral infection was blocked by kaempferol according to the schematics illustrated in Figure 5A. As shown in Figure 5, the co-treatment of CCO cells in the short term failed to decrease viral ORF 39 and ORF 59 mRNA levels, while the virus-treatment and post-treatment of kaempferol significantly reduced the intracellular CCV mRNA level. In addition, we noticed that the viral mRNA level still decreased by approximately 40% when kaempferol was supplemented at 24 h post-infection. Moreover, pre-treatment CCO cells with kaempferol in the short term effectively decreased the intracellular CCV mRNA level at 24 h post CCV infection, and with the prolongation of CCV infection, the inhibitory effect gradually weakened. These results suggest that kaempferol may inhibit CCV entry by targeting either viral particles or host cells.

### Kaempferol inhibited attachment and penetration by CCV

**Figure 6:**
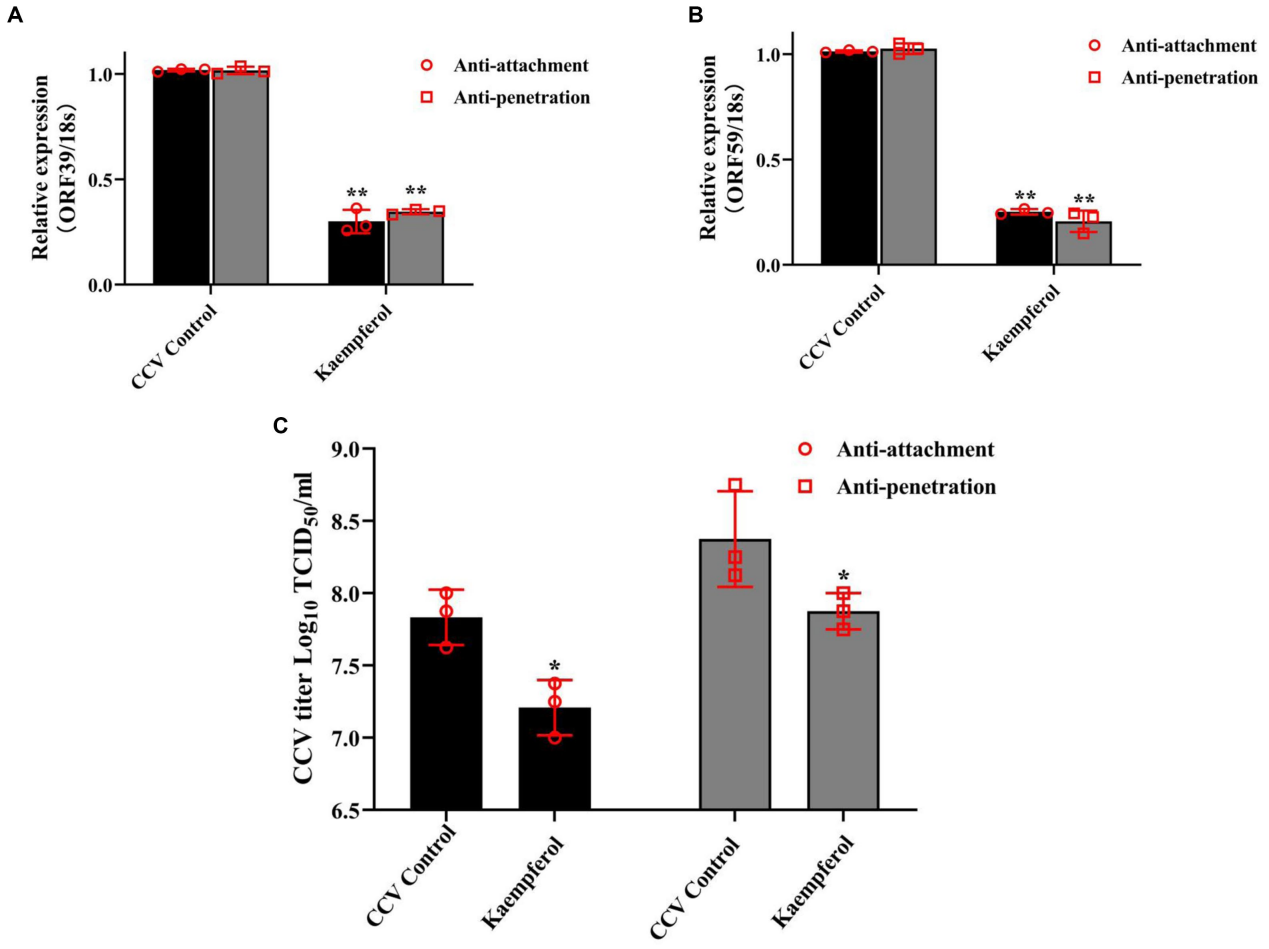
Effects of kaempferol on attachment and penetration analysis. For anti-attachment assay, the CCO cells were pre-cooled at 4°C for 30 min, and then were treated with kaempferol (15 μg/mL) or left untreated for 2 h at 4°C. Then, the cells were infected with CCV at 4°C for 1 h and incubated at 28°C for 24 h. For penetration assay, the CCO cells were prechilled at 4°C, and then infected with CCV at 4°C for 1 h. After triple washing with pre-cooled PBS, the cells were treated with kaempferol (15 μg/mL) or medium at 28°C for 4 h. The compound dilutions were aspirated, the cells were rinsed with citrate buffer (pH 3.0) to inactivate and remove not penetrated virus and incubated at 28°C for 24 h. **(A,B)** The relative expression levels of the CCV ORF 39 and ORF 59 genes in the viral attachment and penetration process. The selected gene level was normalized to 18 s (internal reference) using the 2^− ΔΔCT^ method. **(C)** The determination of viral titer in viral attachment and penetration process. Data represent mean ± SD. The *p* value was determined by *p < 0.05, **p < 0.01.

Attachment and penetration of the virus to the cytomembrane is the indispensable step in viral entry and infection of a cell. To gain further mechanistic insights, virus attachment, and penetration assays were performed in CCO cells. The results of RT-qPCR revealed that exposure to kaempferol significantly down-regulated viral attachment and internalization in host cells by more than 50%, in comparison with the CCV control group (Figures 6A,B). Accordingly, the virus titer in attachment and penetration assays showed that the production of intracellular virus in the kaempferol treatment cells was significantly reduced (Figure 6C). These results suggested that kaempferol was able to block virus entry by reducing the attachment and internalization of viral particles to host cells.

## Discussion

Accompanied by the vigorous global aquaculture industry and the expansion of aquatic products trade, diseases of aquatic organisms continuously appear and cause substantial economic growth losses in aquaculture (36). CCV is the causative pathogen of channel catfish virus disease (CCVD) threatening the channel catfish industry worldwide. Over the past decades, research has mainly focused on the identification, detection, and infection mechanisms of pathogens (37–39). Herein, it is in urgent need of effective prophylactic or therapeutic measures against CCV infection making up for the lack of treatment. In recent years, specifically in the past decade, some studies explored the anti-CCV effects by improving immune response and regulating inflammation (40). Additionally, our previous studies have demonstrated that acyclovir, an acyclic analog of guanosine, exhibits effective anti-CCV activity *in vivo* (33). On account of containing a considerable amount of antimicrobial substances, as well as relatively weak toxicity and comprehensive health benefits in aquaculture, medicinal plants are a potential source for utilization as herbal medications to fight a wide range of pathogenic microbes (41). Here, to seek novel drugs against CCV infection, we preliminary screened several natural compounds for their anti-CCV activity after identifying the safe working concentrations. The RT-qPCR, western blot, and CPE observation results suggested that kaempferol under the maximum tolerated working concentration exhibited potent antiviral efficacy against CCV infection in CCO cells. In the present study, we further evaluated its antivirus ability via RT-qPCR, viral titer determination, western blot, and liquid plaque assay. The results suggested that kaempferol decreased transcription levels of viral genes and the synthesis of viral proteins, as well as reduced proliferation and release of viral progeny, the severity of the CPE induced by CCV in a concentration-dependent manner, which provides valuable information for its future antiviral exploration *in vivo*.

According to the anti-CCV ability exhibited by kaempferol, we further explored its antiviral mechanism *in vitro.* A key step in viral invasion is entry into the host cell requiring the binding of the virus to the cell surface via various specific virus and cell surface proteins (35). Some researchers have reported viral glycoproteins play important roles in the enveloped virus infection (42–44). In our previous study, we found that CCV glycoprotein ORF59 plays a potential role in virus entry into the host cells (7). Additionally, Yu et al. (45) discovered that heparan sulfate interaction with a pH-dependent clathrin-mediated endocytosis pathway mediated the CCV entry into the host cells. Interestingly, most drugs exhibit antiviral activity by disturbing the process of virus infection. It has been confirmed that certain drugs could interfere with virulence via direct target viral proteins, as well as affect host factors to perform their antiviral ability. For example, desoxyrhapontigenin belonging to the polyphenols, could suppress Zika virus entry via dual mechanisms of direct targeting Zika virus E proteins and downregulating putative Zika virus receptors (12). In aquatic animal viruses, quercetin, which has similar chemical molecular structure to kaempferol, interferes with the entry of SGIV to the host cell by disrupting the SGIV protein (46). Artemisinin interferes with spring viremia of carp virus (SVCV) invasion into host cells by impairing the receptors (36). It is well-known that time-of-addition assay can provide a preliminary understanding of the infectious phase upon which the antiviral drug acts. Our study showed that the virus-treatment and post-treatment with kaempferol significantly reduced the viral infection, and pre-treatment in the short term significantly and consistently decreased the intracellular CCV mRNA level. Based on the above results, we speculated that kaempferol might suppress the CCV entry process through interaction with the virus itself or the host cells receptor to reduce CCV infection, although the specific mechanisms involved need further exploration.

Viral attachment and internalization are the key processes for viral entry into the host cells. Many antiviral drugs work by blocking one or more of these steps (47, 48). Antiviral diazadispiroalkane core molecules blocked the attachment of herpesviruses through interaction with sulfated glycosaminoglycans of proteoglycans on host cells (35). Lycorine reduced duck tembusu virus (DTMUV) infection by preventing viral internalization to host cells (49). Coumarins derivative LMC1011 affected the internalization of SVCV but not its attachment (50). Here, we reported that kaempferol exerted its anti-CCV activity by interfering with crucial virus entry steps. Moreover, the anti-attachment and anti-penetration results indicated that kaempferol specifically applied during the attachment and internalization phase significantly reduced viral gene expression and virus titers. Interestingly, we also found that the anti-attachment effect of kaempferol to CCV infection was stronger than the anti-internalization. The above results indicated that kaempferol might interfere with both viral attachment and internalization to achieve the antiviral effect.

In summary, the present study demonstrates that kaempferol exhibits powerful anti-CCV properties *in vitro.* Moreover, our data reveal that kaempferol exerts anti-CCV activity probably by blocking attachment and internalization of the viral entry process. Therefore, our study highlights that kaempferol is a promising and efficient agent against CCV infection, which provides a potential therapeutic option for CCV infection.

## Data availability statement

The original contributions presented in the study are included in the article/Supplementary material, further inquiries can be directed to the corresponding author.

## Ethics statement

Ethical approval was not required for the studies on animals in accordance with the local legislation and institutional requirements because only commercially available established cell lines were used.

## Author contributions

KH: Writing – original draft, Conceptualization, Project administration, Supervision, Funding acquisition. YW: Data curation, Investigation, Methodology, Writing – original draft. JX: Investigation, Writing – review & editing, Methodology. CN: Investigation, Writing – review & editing, Methodology. SS: Investigation, Writing – review & editing, Methodology. FY: Investigation, Writing – review & editing, Methodology. ZZ: Conceptualization, Project administration, Supervision, Writing – review & editing.
