# Supplementary material for: Kaempferol is a novel antiviral agent against channel catfish virus infection through blocking viral attachment and penetration in vitro
Source: Front Vet Sci. 2023 Dec 4;10:1323646. doi: 10.3389/fvets.2023.1323646 (PMC10725991; doi:10.3389/fvets.2023.1323646)
Supplement: Supplementary file 1 [file Table_1.docx]

**Supplementary Material**

Table S1

Purchase information of natural compounds.

| Compound | Cas Number | [purity](https://translate.sogou.com/javascript:%20void(0)) | Purchase from |
| --- | --- | --- | --- |
| Kaemferol | 520-18-3 | 97% | Sigma |
| Tannic acid | 1401-55-4 | 95% | Sigma |
| P-Coumaric acid | 7400-08-0 | 97% | Sigma |
| Magnolol | 528-43-8 | > 98% | Sigma |
| Baicalein | 491-67-8 | ≥ 98% | Sigma |
| Genistein | 446-72-0 | ≥ 98% | Sigma |
| Mangiferin | 4773-96-0 | ≥ 98% | Sigma |
| Rosmarinic acid | 20283-92-5 | ≥ 97% | Sigma |
| Artemisinin | 63968-64-9 | 99% | J&K Scientific |
| Myricetin | 529-44-2 | 97% | Sigma |
| Ginkgolic Acid | 111047-30-4 | ≥ 98% | Sigma |
| Shikonin | 54952-43-1 | 98% | Sigma |
