# Supplementary material for: Kaempferol is a novel antiviral agent against channel catfish virus infection through blocking viral attachment and penetration in vitro
Source: Front Vet Sci. 2023 Dec 4;10:1323646. doi: 10.3389/fvets.2023.1323646 (PMC10725991; doi:10.3389/fvets.2023.1323646)
Supplement: Supplementary file 2 [file Table_2.docx]

**Supplementary Material**

Table S2

The primers used for RT-qPCR

| Primer | Sequences | Product Length(bp) | Access Number |
| --- | --- | --- | --- |
| ORF39-F | GAAGATAGCCCGTCTCACCG | 144 | NC_001493.2 |
| ORF39-R | ATCTCGATCAGCATCTGGCG |  |  |
| ORF59-F | AGGCGTATCACCAACTCACC | 106 | NC_001493.2 |
| ORF59-R | ACCGAACTGGTGAGGATCAG |  |  |
| 18s-F | CGCCCCGCCCAACTCGCCTGAATA | 116 | AF021880.1 |
| 18s-R | CGAATGCCCCCGCCGTCCCTCTTA |  |  |
